# Role of Memory B Cells in Hemagglutinin-Specific Antibody Production Following Human Influenza A Virus Infection

**DOI:** 10.3390/pathogens8040167

**Published:** 2019-09-28

**Authors:** Mark Y. Sangster, Phuong Q. T. Nguyen, David J. Topham

**Affiliations:** David H. Smith Center for Vaccine Biology and Immunology, Department of Microbiology and Immunology, University of Rochester Medical Center, Rochester, NY 14642, USA; phuong_nguyen@urmc.rochester.edu (P.Q.T.N.); david_topham@urmc.rochester.edu (D.J.T.)

**Keywords:** influenza A virus, infection, hemagglutinin, hemagglutin stalk, memory B cells, antibodies, germinal centers, original antigenic sin, imprinting

## Abstract

When influenza A virus infects an immune individual, preexisting memory B cell (MBC) activation and rapid anamnestic antibody production plays a key role in viral clearance. The most effective neutralizing antibodies target the antigenically variable head of the viral hemagglutinin (HA); antibodies against the conserved HA stalk provide broader but less potent protection. In this review, we provide a comprehensive picture of an adult’s HA-specific antibody response to influenza virus infection. The process is followed from preexisting HA-specific MBC activation and rapid production of anti-HA antibodies, through to germinal center seeding and adaptation of the response to novel features of the HA. A major focus of the review is the role of competition between preexisting MBCs in determining the character of the HA-reactive antibody response. HA novelty modifies this competition and can shift the response from the immunodominant head to the stalk. We suggest that antibodies resulting from preexisting MBC activation are important regulators of anti-HA antibody production and play a role in positive selection of germinal center B cells reactive to novel HA epitopes. Our review also considers the role of MBCs in the effects of early-life imprinting on HA head- and stalk-specific antibody responses to influenza infection. An understanding of the processes described in this review will guide development of vaccination strategies that provide broadly effective protection.

## 1. Introduction

B cell memory generated by influenza A virus (IAV) infection and vaccination consists of antibodies (Abs) and memory B cells (MBCs). Preexisting Abs against the virus’s surface glycoproteins, the hemagglutinin (HA) and neuraminidase, have direct antiviral activity and provide the most effective protection against initiation or progression of infection [1,2]. If infection is not blocked or quickly terminated, MBC activation results in rapid and vigorous anamnestic Ab production that acts in concert with other forms of adaptive responses to clear infectious virus [3]. Activated MBCs also contribute to adaptation of the Ab response to novel features of the infecting virus by seeding germinal centers (GCs), where Ab-secreting cells and MBCs with increased binding affinity for IAV antigens are generated [4]. Although the induction of Abs by IAV infection and vaccination has been well-described [5], much less attention has been given to the essential role of MBCs in this process. Here, our goal is to review the Ab response to IAV infection in immune adults with an emphasis on the contribution of MBCs. We consider only IgG-expressing MBCs and focus entirely on the B cell response to the viral HA, the viral attachment protein that initiates cell infection by binding to sialylated receptors [6]. This enables us to consider the response of an MBC pool formed over many years by a series of exposures to related but different HAs through infection and vaccination. HA-intrinsic factors that influence MBC generation include epitope conservation as well as immunodominance hierarchies within the HA molecule [7]. In particular, our review highlights competition between preexisting MBCs and HA novelty as key determinants of the nature of HA-specific Ab production. Our review is primarily based on studies of human B cell responses. However, where appropriate, we incorporate findings from animal models that assist us to develop a more complete picture of processes in responding lymphoid tissues.

## 2. Anti-HA Antibodies: A Brief Overview

Each monomeric component of the homotrimeric HA consists of two structurally distinct domains: a membrane-distal head domain containing the receptor binding site and a membrane-proximal stalk domain [8]. Abs against the HA head that block binding of virus to host cells have the most potent virus neutralizing activity. However, anti-head Abs tend to be virus strain-specific because of the modification of antigenic sites in the head domain by ongoing antigenic drift. Abs against the conserved HA stalk protect via other mechanisms and are less potently neutralizing, but are more broadly reactive across HA variants and subtypes. Two phylogenetic groups of HA subtypes are recognized (group 1 and group 2), with anti-stalk Abs typically cross-reactive within a group. However, across-group stalk-binding Abs have been identified [1,9].

Abs against the IAV HA are also the basis of “original antigenic sin (OAS)” as originally termed [10], and more recently designated “antigenic seniority” [11]. Based on Ab titers measured by hemagglutination inhibition (HAI) assay, which detects Abs that bind near the receptor binding site on the HA head, IAV infection typically generates an Ab response that is broadly HA cross-reactive. Frequently, this response is of the OAS type and characterized by preferential boosting of Abs against sets of HAs related to those of viruses that circulated early in an individual’s life [12,13]. The term OAS has also been applied to baseline circulating HA-reactive Ab levels that are highest against “older” HAs, a pattern that is largely maintained by OAS responses to IAV infection. It is postulated that OAS reflects the lasting imprint of early-life HA exposure, probably in the form of significant IAV infection, on an individual’s immune memory. Mechanistic details of this so-called “imprinting” remain unclear, but patterns of expansion of HA-reactive MBCs are likely to be of central importance as discussed later [14,15]. In addition to imprinting effects on HA-reactive Ab production after IAV infection, the response generally includes adaptation to novel HA features and production of Abs with increased affinity for variant head epitopes [12,13,16]. The HA head domain is immunodominant over the stalk domain and is the target of the vast bulk of HA-reactive Abs produced by IAV infection. However, Abs against the stalk are also generated and, in some situations, can form a strong component of the Ab response to the HA [17].

## 3. MBCs and the Anti-HA Antibody Response to Influenza Infection

### 3.1. MBCs and Initial Antigen Encounter

**Figure 1:**
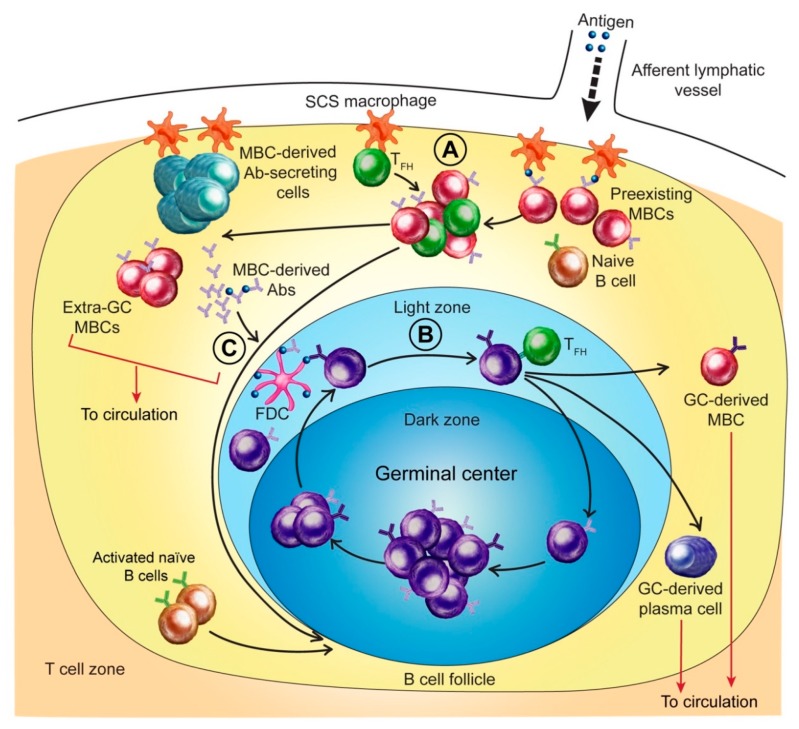
The HA-specific B cell response to seasonal IAV infection in an adult with a typical history of influenza exposure. Results from human and animal studies were used to construct a model of events in a responding lymph node. (**A**) Events in subcapsular proliferative foci (SPF). Influenza proteins (including HA) entering the subcapsular sinus (SCS) are trapped by SCS macrophages. HA-reactive memory B cells (MBCs) beneath the SCS compete for antigen displayed by SCS macrophages. Successful acquisition of antigen enables MBCs to receive cognate T cell help and differentiate along pathways that reflect antigen affinity: high affinity antigen binding (and stronger T cell help) favors formation of Ab-secreting cells; lower affinity cells generate MBCs or seed germinal centers (GCs). (**B**) Events in GCs. GCs are seeded by activated MBCs or activated naïve B cells. After mutation of immunoglobulin V-region genes, GC B cells that express high affinity receptors are positively selected, a process that involves competition for antigen held by follicular dendritic cells (FDCs) so that sufficiently strong T cell help can be secured. Selected cells repeat the mutation/selection cycle or differentiate into MBCs or Ab-secreting plasma cells and exit the GC. (**C**) Regulation by MBC-derived Abs. High affinity HA-reactive MBCs differentiate into Ab-secreting cells in SPF. Secreted Abs potentially regulate anti-HA Ab production by facilitating HA removal or by epitope masking. We suggest that anti-HA Abs released in the SPF enter nearby GCs and bind antigen held by FDCs. Since the initial wave of MBC-derived anti-HA Abs generally binds conserved epitopes with relatively high affinity, masking epitopes on FDC-held antigen would drive selection of GC B cells reactive to the more variant epitopes. Circulating Abs could act in a similar way, but perhaps only after development of lung inflammation increases Ab movement from the vasculature into lung tissue and then to lymph nodes via lymphatic vessels.

B cell activation and the production of Abs against the HA of an infecting IAV begins with transport of the HA (as well as other viral antigens) from the site of infection to local lymphoid tissues and lymph nodes. Antigen binding via the B cell receptor (BCR) and uptake, processing, and presentation to establish cognate interactions with CD4 T cells are essential for MBC and naïve B cell activation [3,18]. This sets the stage for competition for antigen between different types of HA-reactive B cells, for example, between MBCs and naïve B cells, and between MBCs specific for different epitopes on the HA head and stalk. HA-reactive naïve B cells are relatively rare and are vastly outnumbered by HA-reactive MBCs in most adults. After formation, MBCs generally disperse to lymphoid tissues throughout the body [19]. However, MBCs generated by IAV infection are likely to maintain highest frequencies at sites of formation in the respiratory tract [19,20,21]. In addition, localization of MBCs around the periphery of lymphoid tissues or clustered near GC-like structures may allow earlier and more efficient antigen capture and CD4 T cell engagement by MBCs than by the follicle-associated naïve B cells [22,23]. MBCs are also more readily activated than naïve B cells because of cell-intrinsic factors that include epigenetic modifications, altered transcriptional networks, and greater signaling capacity of the IgG BCR [24]. In many cases, it is likely that the Ab response generated by MBC activation after IAV infection is sufficient to terminate infection with little, if any, contribution by naive B cells. Figure 1 provides a diagrammatic overview of processes described in this review.

### 3.2. MBC Activation and Early Anti-HA Antibody Production

Analysis of the Ab response to IAV infection has generally focused on measurement of circulating Abs against the HA of a strain likely to represent the infecting virus, such as the strain in the most recent seasonal influenza vaccine. However, recent studies have emphasized that anti-HA Ab production is characterized by increased titers against a chronological range of HA variants [12,13,16]. Within each individual, the kinetics of the Ab response to each HA variant follows similar kinetics, indicating Ab production at a similar stage of the B cell response. These Abs are first detected in the circulation 4–6 days after symptom onset and increase to peak levels over the next seven days. Ab concentrations against each HA variant form a hierarchy, often reflecting OAS that is largely maintained over the course of the response [13]. Some studies have reported that Ab production is strongest against HAs of the infecting strain and closely related contemporary viruses [12,16]. However, this is the picture only when the response is represented as fold change in Ab titer. The pre-infection level of Abs against the HA of the infecting virus is often low and a relatively modest response to this HA can generate a marked fold increase in Ab levels. Representing the Ab response as the amount of Ab produced (delta for circulating Ab levels) results in a different picture, with strongest Ab production against older HAs [13].

Early production of anti-HA Abs following IAV infection in immune adults results from activation of preexisting MBCs, a model based on analysis of Ab-secreting plasmablasts (PBs) that appear in the circulation at about the same time as the first virus-induced Abs. These PBs generally reach peak frequencies 4–6 days post-symptom onset, but remain detectable for another 1–2 weeks [13,25]. More than 50% of total IgG PBs induced by IAV infection are virus-specific and, of these, up to 50% bind the viral HA [26]. Analysis of the HA-reactive PBs has identified recently proliferated clonal lineages that express Abs with highly mutated immunoglobulin variable genes, indicating derivation from recently activated MBCs that had previously undergone affinity maturation [26].

The site of early MBC-derived PB formation in responding lymphoid tissue has often been imprecisely described as “extrafollicular” [27]. Recently, Moran et al. [28] described a scenario for MBC activation in a skin draining lymph node that might also apply to lung-draining lymph nodes responding to IAV infection. In this scenario, resting MBCs preferentially accumulate in the outer lymph node cortex immediately beneath the subcapsular sinus (SCS) where they are in close association with SCS macrophages that span the floor of the SCS. Large numbers of memory T follicular helper (Tfh) cells occupy the same niche as the MBCs. SCS macrophages efficiently trap antigen carried by draining lymphatics to the SCS and display intact and processed antigen for recognition by memory B and T cells, respectively. Moran et al. [28] describe a structure, designated subcapsular proliferative foci (SPF) that covers the cortical surface of the B cell follicle where it abuts the SCS. Here, SCS macrophages, MBCs, and Tfh cells interact after antigen exposure, resulting in MBC proliferation and differentiation into a cluster of predominately Ab-secreting plasma cells [28,29]. This process likely generates the first HA-reactive IgG Abs produced following IAV infection, as well as the transient wave of circulating HA-reactive PBs. It follows that the SPF is a key site for competition between MBCs.

It is generally accepted that OAS patterns of HA-reactive Ab production after IAV infection reflect the competitive dominance of MBCs reactive to conserved epitopes on the HA head domain, although experimental support for this idea is lacking [14,15]. The MBC pool is established by previous HA encounter, and perhaps most importantly by the stamp of significant early-life infection on a naïve or relatively naïve B cell repertoire. In that situation, the HA-specific B cell response and MBC formation reflects the frequencies of reactive B cells in the unselected repertoire and the immunodominance hierarchy of B cell epitopes on the HA molecule. The severity/duration of early-life IAV infection is also likely to be a key factor in establishing OAS, since that would relate to the establishment and maintenance of GC reactions that expand MBCs and support affinity maturation. A series of subsequent infections by progressively more drifted IAVs would reinforce early imprinting of the B cell repertoire. This process would involve (i) preferential activation (with Ab production and MBC generation) of the more numerous, high affinity MBCs responsive to conserved HA head epitopes and (ii) the potential of anamnestic Ab production to limit infection, thus minimizing adaptation of the B cell response to novel features of the HA of the infecting virus.

**Figure 2:**
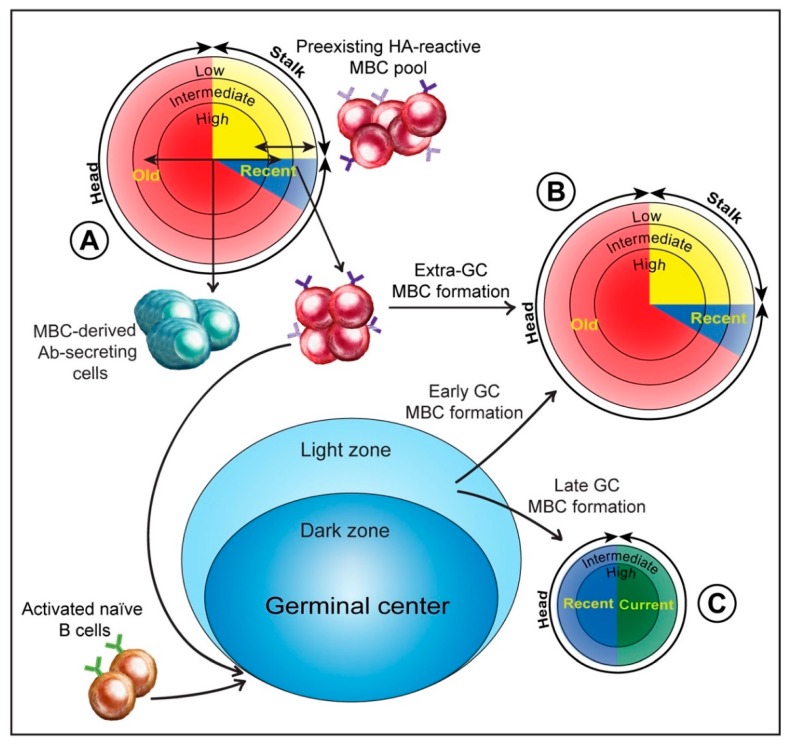
Composition of the MBC pool reactive to the HA of a seasonal IAV at different stages of the response to infection. MBC pools are represented as pie diagrams, with segments representing the proportions of MBCs reactive to HA head domains of older circulating IAVs (“old”; colored red), more recent IAVs (“recent”; blue), and the current circulating IAV (“current”; green), and to the stalk domain (yellow). Concentric circles identify the proportions of MBCs with high (center), intermediate, or low affinity. Composition of the preexisting HA-reactive MBC pool is based on an analysis of the response to a seasonal H3N2 IAV infection [13]; the pathways of formation and the composition of MBC pools generated during infection are speculations based on multiple studies (see Section 3.5 and Section 3.6). (**A**) The preexisting MBC pool reactive to the HA of the infecting virus. Activation and differentiation of cells in this pool via extra-GC pathways generates Ab-secreting cells from high affinity MBCs and MBCs from lower affinity precursors. Activated lower affinity MBCs also seed GCs. (**B**) The MBC pool generated via the extra-GC pathway and early in the GC reaction. Weisel et al. [31] identified an early phase of GC activity that generates primarily MBCs after fewer rounds of mutation and selection. We suggest that this phase, together with the extra-GC pathway, largely reestablishes the preexisting MBC pool. (**C**) MBCs generated by prolonged GC reactions. Although there is evidence that GCs undergo a switch from MBC to plasma cell formation [31], recent studies [32,33] demonstrate that GCs can be long maintained and that the process of MBC adaptation to novel features of an HA is ongoing. We suggest that MBC-derived anti-HA Abs generated early in the response, and perhaps also circulating anti-HA Abs at a later stage, have an important regulatory role and drive positive selection of GC B cells that bind variant HA epitopes (see Figure 1C).

The reactivity profiles of Abs expressed by individual PBs generated early in the response to IAV infection have not been extensively characterized. However, limited studies indicate that at least some of the PB-expressed Abs bind to one or more older HAs, sometimes with higher affinity than to the infecting virus HA [26,30]. These findings are consistent with PB derivation from MBCs that were generated by exposure to older HAs and respond to sufficiently conserved epitopes on the HA of the infecting virus. High affinity MBCs would be expected to outcompete lower affinity MBCs for available antigen and thus obtain the strong level of T cell help associated with differentiation of activated B cells into Ab-secreting PBs. However, it is unclear to what extent antigen affinity or simply the number of MBCs reactive to particular epitopes determines the pattern of early broad HA-reactive Ab production. The importance of MBC affinity might be diminished in the context of IAV infection, which is associated with increased levels of cytokines and Toll-like receptor agonists that help drive B cell activation [27]. Local production of soluble B cell activating factors in the subcapsular niche occupied by MBCs might be particularly important [29]. Figure 2 shows a representation of a preexisting HA-reactive MBC pool that responds to IAV infection; outcomes of MBC activation and their relationship to HA affinity are also shown. Pathways of MBC formation that we suggest maintain and/or modify the preexisting MBC pool are included in Figure 2 and are discussed later.

### 3.3. MBCs and Anti-HA Stalk Antibody Production

In addition to the broad HA head-reactive Ab response, approximately 10%–20% of anti-HA Abs generated by seasonal IAV infection bind to the stalk domain. The response to the stalk follows the same kinetics as the anti-head response and includes early induction of stalk-specific PBs with high numbers of mutations in immunoglobulin variable genes, indicating formation from activated MBCs [13,26]. A key determinant of the magnitude of the Ab response to the stalk is the dominance of head-reactive over stalk-reactive MBCs in competition for antigen. An individual’s exposure to HA through vaccination and infection would be expected to progressively increase the ratio of head- versus stalk-reactive MBCs, primarily because of immunodominance of the head. In addition, the relatively small year-by-year changes in the HA head through antigenic drift would enable recruitment and expansion of similar head-reactive MBCs over many HA exposures. HA stalk-reactive MBCs are expanded by infection in parallel with head-reactive MBC [13]. However, evidence that stalk-binding Abs display a level of polyreactivity raises the possibility that stalk-reactive MBCs might be selected against by tolerance mechanisms [34]. Notably, few stalk-reactive Abs are produced after seasonal inactivated influenza vaccination [26,30], suggesting that factors associated with IAV infection facilitate stalk-reactive MBC activation. These might include the amount and form of the HA presented to MBCs, together with a B cell activating environment associated with infection that reduces the requirement for T cell help [27].

Studies of responses to infection and vaccination with the 2009 pandemic H1N1 (pH1N1) IAV emphasize the importance of competition between MBCs in determining the nature of HA-specific Ab production. The H1 of the pH1N1 IAV was substantially novel to most individuals less than approximately 60 years of age when the virus began circulating in humans. Initial exposure of these individuals to the pH1N1 virus through infection or vaccination generated anti-HA Abs that were broadly cross-reactive with head and stalk epitopes of multiple influenza strains [26,35,36]. In particular, the strong anti-stalk response to pH1N1 vaccination contrasted with the almost entirely head-directed response after seasonal influenza vaccination. It is now appreciated that strong anti-stalk Ab production after pH1N1 infection or vaccination reflects the novelty of the HA head domain [35]. As a result, numbers (and/or affinities) of preexisting MBCs reactive with the pH1 head are reduced, competition with stalk-reactive MBCs is lessened, and the anti-stalk Ab response is increased. The scenario is very similar when individuals are infected or vaccinated with avian IAVs, such as the H5N1 and H7N9 subtypes, which express head domains that are highly novel to most humans and stalk domains that are strongly conserved with those of seasonal IAVs. Responses to novel avian IAV HAs in adults are characterized by strong production of anti-stalk Abs, reflecting activation of preexisting stalk-reactive MBCs largely in the absence of competition from head-reactive MBCs [37,38]. Notably, the anti-HA response to avian H7N9 IAV infection consists of an early phase of broadly reactive anti-stalk Ab production, followed later by production of HAI-mediating Abs against the H7 head [39]. This pattern likely reflects early stalk-reactive MBC activation, then formation and maintenance of GC reactions that support affinity maturation of head-reactive B cells. These could include naïve B cells recruited into the response and low affinity MBCs that were not driven to PB formation. Apparently, the potent immune stimulus provided by avian IAV infection increases GC output, resulting (eventually) in production of efficiently neutralizing anti-HA head Abs that are not generated by a single dose of avian influenza vaccine [40].

### 3.4. MBCs and Imprinting of Antibody Responses to the HA Stalk

An effect of early-life imprinting on the character of an individual’s Ab response to the HA head following IAV infection is well-established [13], but it is less clear whether responses to the stalk are also impacted. A possible scenario is that the first influenza subtype (H1N1 or H3N2) responsible for significant early-life infection imprints (through MBC expansion) not only a pattern of responsiveness to HA head epitopes, but also the potential to respond strongly to group 1 (e.g., H1) or group 2 (e.g., H3) HA stalk domains. In support of this is evidence that stalk-specific B cell lineages are established early in life and are determined in part by the subtype of the first infecting IAV [41]. Patterns of anti-stalk Ab production consistent with imprinting are unlikely to be evident in the response to seasonal IAV infection. This is because of the competitive dominance of HA head-reactive MBCs and the major role of anti-head Abs in determining the course of infection) [42]. However, the situation is very different when the HA of the infecting virus is highly novel and early anti-HA Ab production is essentially dependent on activation of preexisting MBCs reactive to the conserved stalk. This is the situation following avian IAV infection, where recovery might depend on the magnitude of anti-stalk production. Notably, Gostic et al. [43] provided evidence that individuals imprinted by a first infection with an H1N1 (group 1 HA) or an H3N2 (group 2 HA) IAV have a less severe disease when infected as adults with the avian IAVs H5N1 (group 1 HA) or H7N9 (group 2 HA), respectively. Possible explanations are higher baseline levels of circulating anti-stalk Abs or larger MBC populations reactive to the stalk of the avian HA. Recently, Tesini et al. [13] provided evidence that group 2 HA stalk-specific MBC populations expanded by seasonal H3N2 (group 2 HA) infection were largest in individuals imprinted by early-life H3N2 infection; the same was not true for group 2 HA stalk-specific Ab levels. This suggests that the imprinting effects described by Gostic et al. [43] are mediated by expanded stalk-reactive MBC populations that cross-react with either the group 1 or group 2 HA stalks of heterosubtypic IAVs.

### 3.5. Extra-Germinal Center Generation of MBCs

The pathways that maintain or expand HA-reactive MBC populations after IAV infection are not fully understood. For instance, it is unclear whether preexisting IgG MBCs activated in the SPF generate both PBs and MBCs via extra-GC pathways or whether MBCs are only generated in GCs after some level of remodeling of antigen reactivity. In the absence of GCs, naïve B cell activation can produce IgG MBCs that have not undergone affinity maturation [44,45], raising the possibility of an extra-GC pathway of MBC formation that maintains/expands IgG MBC populations activated to generate the early PB response (see Figure 2). In line with this idea is the observation that recently proliferated, HA-reactive MBCs increase in the circulation 4–6 days after onset of symptoms post-IAV infection, closely accompanying the initial increase in HA-reactive PBs and Ab levels [13,46]. Notably, the recent analysis by Tesini et al. [13] demonstrated a close relationship between patterns of early Ab production after IAV infection measured against a range of HA variants (including OAS patterns) and patterns of early expansion of MBC populations reactive to the same set of HAs. This result is consistent with Ab production and MBC formation stemming from activation of the same precursor MBC. B cell clonal lineage analysis by Ellebedy et al. [46] also indicated that PB and MBC lineages could have originated from the same activated MBC, but does not exclude a contribution of GCs to at least part of the MBC lineage.

### 3.6. Germinal Center Events

A subset of HA-reactive B cells activated by IAV infection enter GC reactions where they undergo affinity maturation [47]. A proportion of the preexisting MBCs activated by infection become GC B cells as an alternative to extra-GC differentiation into PBs or MBCs. Activated naïve B cells might also enter GCs, especially when infection is severe and the response is prolonged [26]. A key determinant of the fate decision of an activated naïve or memory B cell is affinity for antigen, with high affinity linking directly to strong T cell help and favoring Ab-secreting cell formation [48,49]. Entry of activated cells into GCs was associated with an “intermediate” level of T cell help [50]. However, the threshold affinity requirement for GC seeding could be modulated by increased levels of B cell activating factors in the infection-associated environment, resulting in GC entry of low affinity B cells that might otherwise have formed MBCs or been lost from the response [3,27,51,52].

After Ab V-region somatic hypermutation, GC B cells that express BCRs with high antigen affinity are positively selected for further cell division and mutation or for differentiation along pathways that generate plasma cells or MBCs. The process of positive selection is not fully understood, but signaling to GC B cells via the BCR and CD40 plays a central role. A key step is competition between GC B cells for antigen displayed on the surface of follicular dendritic cells (FDCs); Ag that is bound with sufficient affinity by GC B cells is taken up, processed, and presented to secure cognate help from Tfh cells [53]. Importantly, Tas et al. [54] provided evidence that GCs can be seeded by a highly oligoclonal pool of activated B cells (estimated at 10 s to 100 s of clones per GC). This sets the stage for a combination of two types of competition between GC B cells within single GCs: intraclonal competition between mutational variants of the same clone (specific for the same epitope), and interclonal competition between clones specific for different epitopes on the same or different antigens. In addition to numbers and affinities of specific GC B cells and antigen abundance and form on FDCs, antigen-intrinsic immunodominance hierarchies might also play an important role in determining the outcome of interclonal competition in GCs that (for example) drive selection of HA head-reactive over stalk-reactive GC B cells.

Recent studies of human responses to avian HAs, including H5 expressed by a replicating adenovirus, provide evidence that GC activity continues for many months after immunization [32,33]. It is, therefore, reasonable to expect long-lived GC reactions after seasonal IAV infection, allowing factors such as affinity-matured Abs or a general reduction in available antigen to continue to influence selection of GC B cells and drive affinity maturation. The signals that direct selected GC B cells along the plasma cell or MBC differentiation pathways before exiting the GC are similar to those responsible for extra-GC fate decisions. Generally, high affinity GC B cells that acquire antigen efficiently and receive strong T cell help form plasma cells; lower affinity B cells enter the MBC pool [47]. Experiments in mice indicate that GCs initially generate mostly MBCs and then switch after two or more weeks to plasma cell formation [31]. MBC formation after fewer rounds of selection would limit the extent of adaptation to the HA of the infecting virus, but might provide the advantage of expanding MBCs with greater breadth of reactivity. The temporal switch in GC output from MBCs to plasma cells fits with ongoing cycles of mutation and selection and the preferential formation of plasma cells from B cells expressing high affinity receptors. There is nevertheless a degree of affinity maturation of MBCs generated in GCs. Tesini et al. [13] analyzed responses to seasonal H3N2 infection in the 2012–2013 season when infecting viruses were HA drift variants [55]. Circulating MBCs adapted to the H3 head domain and perhaps generated during the early phase of MBC production in GCs were detected within approximately four weeks of symptom onset. However, evidence that HA-reactive MBCs with increased levels of somatic hypermutation are formed many months after HA exposure indicates that affinity maturation of MBCs continues long after a switch by GCs to predominantly plasma cell production (see Figure 2) [32,33].

### 3.7. Regulation of the Anti-HA Response by MBC-Derived Antibodies

Preexisting anti-HA Abs and anti-HA Abs generated at various stages after IAV infection are thought to play an important role in regulating the fine specificity of the anti-HA Ab response [15]. The anti-HA Abs could block B cell recognition of particular epitopes/antigenic sites by epitope masking or more generally suppress the response by facilitating HA removal through Fc-mediated mechanisms [56,57]. Epitope masking by preexisting Abs does not fit with the observation that patterns of HA-reactive Ab production early in the response to IAV infection directly reflect OAS hierarchies of HA-reactive Ab levels in the circulation prior to infection [13]. An important consideration is whether or when preexisting circulating Abs have sufficient access to sites of immune response generation to exert a regulatory influence. For instance, circulating Abs might always have ready access to events in the spleen, but might not reach high levels in lymph nodes responding to IAV infection until anti-viral responses commence in the respiratory tract and extend to draining lymph nodes [58,59]. We suggest that preexisting circulating Abs have little if any effect on MBC activation in the SPF of local lymph nodes; this component of the response generates the first anti-HA Abs and is primarily regulated by MBC competition. Furthermore, we speculate that Abs resulting from early MBC activation in the SPF play a key role in response regulation by mechanisms like epitope masking. Perhaps most important is the potential for these Abs to regulate positive selection in GCs, a possibility suggested by the close proximity of the SPF and B cell follicles. Free Abs might enter GCs and interact with antigen on FDC networks, or bind antigen outside GCs and enter as immune complexes. Zhang et al. [60] demonstrated that Abs enter GCs, bind antigen held on FDCs, and compete with GC B cells in an affinity-dependent way. Through these mechanisms, Abs derived from preexisting MBCs could facilitate affinity maturation of responses to drifted epitopes on the HA head [61]. In the case of MBC-derived anti-stalk Abs, GC B cell selection would be shifted towards targeting the immunodominant HA head [62].

## 4. Summary

Our goal in this review is to combine observations from human and animal studies to provide a picture of the HA-specific B cell response to IAV infection in adults. A critical determinant of the nature of the response in adults is the HA-specific B cell memory established by HA exposure through IAV infection and vaccination over many years. Of central importance is the composition of an individual’s HA-reactive MBC pool, a population that even in adulthood reflects the imprint of early-life HA exposure and is responsible for OAS patterns of Ab production early in the response to IAV infection. PB formation from activated preexisting MBCs with high affinity for the HA of the infecting virus optimizes the protective efficacy of the initial wave of anti-HA Abs. We emphasize MBC competition as a key determinant of the patterns of activation of preexisting MBCs. The novelty of the HA of the infecting virus has a profound influence on the outcome of MBC competition and (for example) determines the strength of Ab production against the HA stalk. Preexisting MBC activation generates cells that along with activated naïve B cells, seed GCs for generation of MBCs and plasma cells adapted to variant/novel HA epitopes. This process involves another form of B cell competition, the competition between GC B cells for antigen held by FDCs; acquisition of this antigen is a prerequisite for positive selection. We suggest that Abs resulting from preexisting MBC activation, more so than preexisting circulating Abs, play an important regulatory role in positive selection through epitope masking. Depending on the epitope specificity of Abs derived from preexisting MBCs, GC B cells will be refocused on the immunodominant HA head or on particular drifted head epitopes. Our review also considers the generation and expansion of MBCs at different points in the response to both maintain OAS response patterns and adapt to emerging HAs. Our increasing understanding of MBC competition, immunodominance hierarchies, and Ab regulation of B cell responses will continue to guide development of vaccine composition and administration strategies to optimize B cell-mediated protection generated against IAV infection [63,64,65,66,67].
